# Nasal Spray Formulations Based on Combined Hyalurosomes and Glycerosomes Loading *Zingiber officinalis* Extract as Green and Natural Strategy for the Treatment of Rhinitis and Rhinosinusitis

**DOI:** 10.3390/antiox10071109

**Published:** 2021-07-11

**Authors:** Eleonora Casula, Maria Letizia Manca, Matteo Perra, Jose Luis Pedraz, Tania Belen Lopez-Mendez, Antonio Lozano, Esteban Calvo, Marco Zaru, Maria Manconi

**Affiliations:** 1 Department of Scienze della Vita e dell’Ambiente, University of Cagliari, via Ospedale 72, 09124 Cagliari, Italy; e.casula@studenti.unica.it (E.C.); Matteo.perra@unica.it (M.P.); manconi@unica.it (M.M.); 2 NanoBioCel Group, Laboratory of Pharmaceutics, School of Pharmacy, University of the Basque Country (UPV/EHU), 01006 Vitoria-Gasteiz, Spain; joseluis.pedraz@ehu.eus (J.L.P.); tblopez01@gmail.com (T.B.L.-M.); 3 Biomedical Research Networking Center in Bioengineering, Biomaterials and Nanomedicine (CIBER-BBN), 01006 Vitoria-Gasteiz, Spain; 4 Bioaraba, NanoBioCel Research Group, 01006 Vitoria-Gasteiz, Spain; 5 LIFTEC, CSIC—Universidad de Zaragoza, María de Luna 10, 50018 Zaragoza, Spain; a.lozano@csic.es (A.L.); calvober@unizar.es (E.C.); 6 Icnoderm Srl, Sardegna Ricerche Ed. 5, Pula, 09010 Cagliari, Italy; m.zaru@icnoderm.com

**Keywords:** *Zingiber officinalis*, traditional medicine, phospholipid vesicles, antioxidant, epithelial cells, nasal spray, spray angle, droplet size

## Abstract

A total green nanotechnological nasal spray has been manufactured and proposed as an alternative treatment of rhinitis and rhinosinusitis. It was obtained by combining the strengthening effect of liposomes on barrier function, the hydrating and lubricating properties of sodium hyaluronan and the anti-inflammatory and antioxidant activities of the extract of *Zingiber officinalis*. To this purpose, the extract was loaded in special phospholipid vesicles immobilized with hyaluronic acid (hyalurosomes), which were further enriched with glycerol in the water phase. Liposomes and glycerosomes were prepared as well and used as reference. Vesicles were oligolamellar and multicompartment, as confirmed by cryogenic transmission electron microscopy (cryo-TEM) observation, small in size (~140 nm) and negatively charged (~−23 mV). Spray characteristics were evaluated by using the Spraytec^®^ and instant images, from which the plume angle was measured. The range of the droplet size distribution and the narrow spray angle obtained suggest a good nebulization and a possible local deposition in the nasal cavity. In vitro studies performed by using human keratinocytes confirmed the high biocompatibility of vesicles and their ability to effectively counteract oxidative damage on cells induced by hydrogen peroxide. The overall collected data suggest that our vesicles are suitable as nasal spray.

## 1. Introduction

Rhinitis and rhinosinusitis are the two major clinical pathologies involving the upper airways [1]. They differ in their atopic status, symptom severity, duration, medical history and aetiology but have some common clinical presentations such as mucosa inflammation, sneezing, pruritus, purulent rhinorrhoea, nasal congestion, facial pressure and loss of smell. In addition, hyperresponsiveness to nonallergic stimuli, such as cold air and irritants is usually present due to the inflammation on the sensory nerves in the upper airway mucosa [2]. Rhinosinusitis involves inflammation of paranasal sinuses, other than the nasal cavity mucosa. Allergic rhinitis is commonly assumed to have a cause and effect relationship with chronic rhinosinusitis [3]. They are serious common disorders that affects between 15% and 45% of the population worldwide negatively influencing the daily life of patients, especially those with severe symptoms and major disabilities [4].

The recommended treatment requires a combination of oral therapy and intranasal administration of corticosteroids, anti-leukotrienes and antihistamines to reduce mucosal oedema and promote sinus drainage, and eradicate infections in chronic rhinosinusitis [5]. In addition, nasal irrigation has been proven to be an effective additional treatment. It can reduce the associated symptoms and the frequency of acute exacerbation [6,7]. This treatment directly enhances the movement of the mucus toward the nasopharynx, removes inflammatory mediators such as histamine, prostaglandins and leukotrienes, and increases the ciliary beating frequency and consequently the mucociliary clearance [8]. Hyaluronic acid is widely used in allergic or non-allergic rhinitis and acute or moderate rhinosinusitis to reduce disease symptoms, neutrophil count and improve mucociliary transport [9]. It is a typical component of normal airway secretions, which hydrates and lubricates the mucosae of the upper respiratory tract by osmotic effect. It is also involved in the regulation of vasomotor tone and gland secretion and stimulates mucociliary clearance. In addition, hyaluronic acid can regulate inflammatory response by immune-suppressing role [10].

Liposomes are also used as nasal spray in rhinitis to support the cleansing, lubrication and hydration of nasal mucosa. Several studies demonstrated their effectiveness in reducing the symptoms of rhinitis probably because phospholipid are natural occurring surfactants, which reinforce the protective nasal layer [11]. Liposomal nasal sprays exert an effect comparable to that of antihistamine and glucocorticosteroid sprays [12,13]. Indeed, in the German pharmaceutical market, liposomal nasal sprays have been available since 2007 for the treatment of inflamed nasal mucosa [14]. The exact mechanism of action of liposomes is still unknown. It was hypothesized that they stabilise the nasal mucosal barrier by integrating in the damaged cell membrane and strengthening their barrier function [15]. Other studies disclosed that liposomes can absorb and inactivate allergens in allergic rhinitis [16,17].

Several herbal sprays have been used in the local treatment of rhinitis or rhinosinusitis due to their anti-inflammatory and anti-oxidant properties [18]. Ginger (*Z. officinalis*) is widely used as a spice in the world but it is also known for its beneficial properties [19]. Historical evidences attest that the therapeutic efficacy of ginger inhalation was already known since 4000 years in India where it was used for the treatment of respiratory diseases [20,21]. At the dawn of medical and pharmacological science, the administration of chemicals by local inhalation is consistent, because it is the most patient-friendly, natural, spontaneous and painless rout of administration. Today, the inhalation of ginger-based drugs is still used, mainly to counteract nausea and vomiting in post-surgery patients [22,23,24]. According to Stappen et al., the main effect of ginger inhalation is achieved by the odour [25]: the inhalation combines the effect given by the ginger’s active compounds and its pungent smell, making it as a good alternative for the treatment of infectious diseases of the upper respiratory tract as well. Recent research proved that ginger is an effective remedy against respiratory diseases, such as cough, cold and asthma [26,27], acting as antibacterial [28,29], anti-inflammatory [30,31] and muscle relaxant [32] and in vivo experiments confirmed the bronchodilator effect of ginger on histamine-induced bronchospasms [33,34,35].

In this work we combined the effectiveness of liposomes and hyaluronic acid with the anti-inflammatory and antioxidant properties of ginger to manufacture nanotechnological nasal sprays suitable for the treatment of both rhinitis and rhinosinusitis. Indeed, the extract was loaded in special phospholipid vesicles immobilized with hyaluronic acid (hyalurosomes) and further improved with glycerol. Liposomes and glycerosomes were prepared as well and used as reference. The vesicle formation and morphology along with their size and zeta potential were measured. The ability of sprayed dispersions to reach the anterior nasal cavity was fully evaluated along with the biocompatibility of these systems towards keratinocytes and the ability to counteract oxidative damage induced in cells by using hydrogen peroxide.

## 2. Materials and Methods

### 2.1. Materials and Reagents

Lipoid S75 (consisting of ~70% of soy phosphatidylcholine, 9% phosphatidylethanolamine and 3% lysophosphatidylcholine) was purchased from Lipoid GmbH (Ludwigshafen, Germany). Powder extract containing 5% of *Z. officinalis* was purchased by Farmalabor Srl (Italy). Sodium hyaluronate with low molecular weight (200–400 kDa) and a polydispersity of 1.4 Mw/Mn, was purchased from DSM Nutritional Products AG Branch Pentapharm (Switzerland). Glycerol, DPPH radical (2,2-diphenyl-1-picrylhydrazyl), mucin from porcine stomach and all other reagents of analytical grade were purchased by Sigma-Aldrich (Milan, Italy).

### 2.2. Vesicle Preparation

The extract of *Z. officinalis* (30 mg/mL) was blended with phospholipid S75 (90 mg/mL) and dispersed in different aqueous mixtures. To obtain liposomes, a blend of water (900 µL) and PEG400 (100 µL) was used as hydrating medium; to prepare hyalurosomes, sodium hyaluronate (0.1%) dispersed in water (900 µL) and PEG400 (100 µL) were used; to obtain glycerosomes, a blend of water (800 µL), PEG400 (100 µL) and glycerol (100 µL) was used; to prepare glyhyalurosomes, a dispersion of sodium hyaluronate (0.1%) in water (800 µL), PEG400 (100 µL) and glycerol (100 µL) were used. The dispersions were sonicated (25 cycles 5 on/2 off, amplitude 13 µ) using a Soniprep 150 sonicator (MSE Crowley, London, UK) to obtain homogeneous dispersions with small vesicles. Empty vesicles without *Z. officinalis* extract were prepared as well and used as reference. After preparation, vesicles were stored at 4 °C.

### 2.3. Morphological Analysis Using Cryo-TEM

The vesicle morphology was observed by cryo-TEM using a Tecnai G2 20 Twin (FEI), operating at an accelerating voltage of 200 KeV in a bright-field image mode and low-dose image mode. An aliquot of sample (3 μL) was applied to glow-discharged 300 mesh Quantifoil TEM grids and the excess of water was removed with a filter paper. The prepared sample was frozen with a plunge freezing into liquid ethane on a FEI Vitrobot Mark IV (Eindhoven, The Netherlands) to preserve the sample in a frozen solid state. The frozen grids were then transferred to a 626 DH Single Tilt Cryo-Holder (Gatan, France), where it was maintained below −170 °C and then transferred to TEM at liquid nitrogen temperature (−196 °C).

### 2.4. Vesicle Characterization

Light scattering technology was used to determine the average diameter, polydispersity index and zeta potential of designed vesicles by using a Zetasizer Ultra (Malvern Instruments, Worcestershire, UK). These parameters were estimated over a storage period of 10 months at 4 °C to evaluate vesicle long-term stability.

Each sample (2 mL) was purified from the non-incorporated extract by dialysis (Spectra/Por^®^ membranes, 3 nm pore size; Spectrum Laboratories Inc., Rancho Dominguez, CA, USA) in water (2:1) at room temperature for 2 h (refreshing the water after 1 h). The dialysis method has been chosen as the main components of the extract are polyphenols having a molecular weight lower than the cut-off of the membrane (Spectrapor, 12–14 kD), thus they can pass through the membrane.

The antioxidant activity of formulations was measured by the DPPH (2,2-diphenyl-1-picrylhydrazyl) colorimetric test, before and after the dialysis process. Each formulation (20 µL) was dissolved in 1980 µL of DPPH methanolic solution (40 µg/mL). The methanolic solution of DPPH at the same dilution (1:50) was used as a control (100% absorbance). Samples were incubated for 30 min at ~25 °C in the dark. Then, the absorbance of each solution was measured at λ = 517 nm by using a UV spectrophotometer (Lambda 25, Perkin Elmer, Milan, Italy). All the experiments were performed in triplicate. The antioxidant activity was calculated as percentage according to the following formula [36,37]:

(1) $$\mathrm{Antioxidant}\;\mathrm{activity}\;\left(\%\right)=\frac{\left[\left({\mathrm{ABS}}_{\mathrm{DPPH}}-{\mathrm{ABS}}_{\mathrm{sample}}\right)\right]}{{\mathrm{ABS}}_{\mathrm{DPPH}}}\times 100$$

The entrapment efficiency of the vesicles was expressed as the percentage of the antioxidant activity after dialysis versus the value obtained before dialysis.

**Figure 1:**
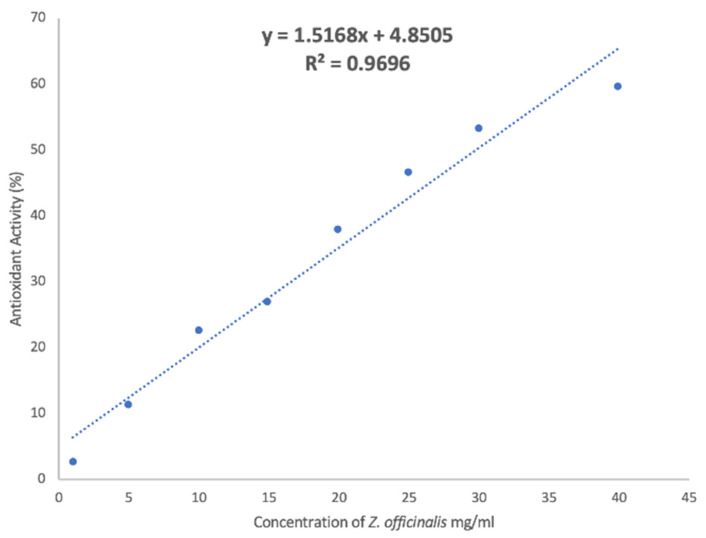
Calibration curve of the antioxidant activity of *Z. officinalis* extract as a function of its concentration.

To evaluate the concentration of the extract before and after dialysis, a calibration curve has been built by reporting their antioxidant activity as a function of the concentration (from 1 to 40 mg/mL) of the extract (Figure 1).

### 2.5. Determination of Droplet Size Distribution

Droplet size distribution was evaluated by laser diffraction using a Malvern Spraytec^®^ (Malvern Panalytical Ltd., Malvern, UK). Formulations (6 mL) were loaded in commercial pump devices (20 mL) kindly supplied by FAES laboratories. In accordance to FDA recommendations (laser distance range must be comprised between 2 and 7 cm with a difference of 3 cm between each different experiment) [38,39], measurements were performed in triplicate at 25 °C, at 4 cm and 7 cm of distance from the nozzle exit, rotating the pump device 45° respect to the laser beam [40].

Data were reported as the D10, D50, D90 volume diameter percentiles, i.e., 10%, 50% and 90% of the cumulative volume undersize. The distribution Span defined as (D90-D10)/D50 was also reported to characterize its width.

### 2.6. Spray Structure, Drop Average Velocity Module and Spray Angle Measurements

The average velocities of the sprayed droplets were determined by Particle Image Velocimetry and the spray opening angle by laser plane visualization. Particle Image Velocimetry method allows to capture two components of the velocity with high spatial resolution in a whole slice of the flow measuring the displacement of the droplets between two sequential images acquired with a known time interval (∆*t*). Instantaneous images were acquired with a Hamamatsu 1024 × 1344 pixels 12-bit C4742-95-12 ORCA-ER charge-coupled device camera (Hamamatsu Photonics, Shizuoka, Japan). To freeze the motion, a PILS Nd:YAG laser from Quanta System (Quanta System, S.p.A., Milan, Italy) capable of generating 6 ns pulses with a maximum energy of 80 mJ at 532 nm was used as a light source capable of illuminating a vertical plane across the center of the spray. The camera was located perpendicular to the illuminated plane, to avoid geometrical distortions. The PILS system has two laser oscillators, so it can generate pairs of pulses with a selectable interval between them. The time interval between the two images in each pair was set at 30 μs. Image pairs were processed with the CCDPIV computer code developed at the Laboratory for Turbulence Research in Aerospace and Combustion (LTRAC) in Monash University (Melbourne, Australia) as described in [41]. Analysis was performed in 32 × 32 pixel windows with 50% overlap resulting in maps with 82 × 62 velocity vectors. 100 instantaneous velocity measurements were performed to obtain the mean velocity field and they were averaged to determine the angle of the spray cone. This angle was obtained by the ocular location of the limits of the spray in the average image. All photographs were taken under the same light conditions and underwent the same renormalization of the light levels captured by the camera. To eliminate the influence of the operator in the generation of the spray, a pneumatic device for driving the manual atomizer was constructed. The duration of the atomization pulse varies according to the supply pressure of the pneumatic device. This pressure has been adjusted to obtain a reasonable actuation, with a pulse duration of 150 ms as measured from the images. The spray speed and angle measurements were taken 83 ms after the start of the atomization, that is, about 8 ms after the mid-pulse.

Finally, the commercial atomizers used were manufactured with plastic injection molding. This technique does not guarantee a high dimensional quality, so a certain variability in the atomization of each device could be expected. To evaluate this variability, distilled water was atomized in triplicate (w1, w2 and w3) as reference under the same conditions used for the pharmacological formulations. Commercial atomizers manufactured with plastic injection molding were used. To evaluate the variability in the atomization of each device, distilled water was atomized in triplicate (w1, w2 and w3) as reference, under the same condition of the samples.

### 2.7. Biocompatibility of Extract Loaded Vesicles against Keratinocytes

Human epidermal cells (HaCaT) were grown as monolayers in 75 cm^2^ flasks, incubated with 100% humidity and 5% CO_2_ at 37 °C. Dulbecco’s Modified Eagle Medium (DMEM) with high glucose, supplemented with 10% fetal bovine serum and penicillin/streptomycin, was used to culture keratinocytes. The cells were seeded into 96-well plates at a density of 7.5 × 10^3^ cells/well and after 24 h of incubation, were exposed for 24 h to the extract loaded vesicles properly diluted to reach different concentrations (0.3, and 3 μg/mL). The extract dispersed in water at the same dilutions was used as reference. The possible toxic effect of the formulations towards HaCaT cells was assessed by measuring cell viability by the MTT (tetrazolium salt, 3-(4,5-dimethylthiazol-2-yl) -2,5-diphenyltetrazolium bromide) colorimetric test. The reduction of MTT by mitochondrial dehydrogenase leads to the formation of crystals of a blue-violet formazan and allows the estimation of the number of living cells in culture.

MTT (100 µL) was added to each well and incubated at 37 °C for 2/3 h. The formazan crystals formed in viable cells were dissolved in DMSO, and the absorbance measured at λ = 570 nm by using a microplate reader (Synergy 4, BioTek Instruments, AHSI S.p.A, Bernareggio, Italy). All the experiments were repeated at least three times and each time in triplicate. The results are expressed as the percentage of viable cells compared to untreated cells (100% viability).

### 2.8. Protective Effect of the Extract Loaded Vesicles against Oxidative Damages Induced in Cells

HaCaT cells (5 × 10^4^ cells/well) were seeded in 96-well plates with 250 μL of culture medium and incubated at 37 °C for 24 h. Then, cells were stressed with hydrogen peroxide (1:50.000) and immediately treated with different concentrations of the extract in dispersion or loaded into vesicles (3, 0.3 µg/mL). Unstressed cells were used as negative control (100% viability); cells stressed with hydrogen peroxide and treated with medium without extract were used as positive controls. After 4 h of incubation the medium was removed and the viability of the cells was determined with the MTT colorimetric test, adding 100 μL of reagent in each well. After 2/3 h, the formed formazan crystals were solubilized by adding DMSO and their absorbance was measured spectrophotometrically at λ = 570 nm.

### 2.9. Statistical Analysis

The results were expressed as mean value ± standard deviation. Statistically significant differences were determined using variance analysis, ANOVA, Test T and Test F. The minimum level of significance chosen was *p* < 0.05.

## 3. Results

### 3.1. Vesicle Characterizations

**Figure 2:**
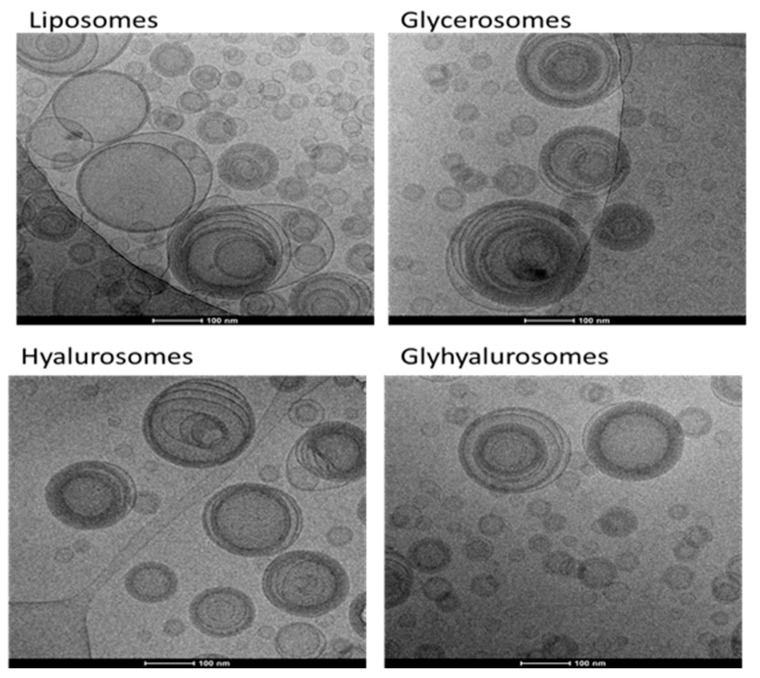
Representative images of liposomes, glycerosomes, hyalurosomes and glyhyalurosomes.

The actual formation of lamellar vesicles and their morphology and structure were evaluated by means of cryo-TEM observation (Figure 2). Vesicles were spherical and not highly homogeneous in size. Indeed, a large amount of very small oligolamellar vesicles was observed along with some larger multilamellar and sometimes multicompartment vesicles.

To complete the vesicle characterization, mean diameter, polydispersity index, zeta potential and entrapment efficiency were measured (Table 1). Empty vesicles were also prepared and characterized as well to evaluate the effect of the extract loading on the vesicle assembling. Empty liposomes were the smallest (~92 nm) while empty hyalurosomes the largest (~175 nm) and both disclosed the more negative values of zeta potential (~−60 mV). The loading of the *Z. officinalis* extract allowed an increase of the size of liposomes (~151 nm, *p* < 0.05 versus the size of corresponding empty vesicles) probably due to the interaction of extract components with the surface of the phospholipid bilayer, which in turn change the curvature radius of vesicle membrane. Result was confirmed by their zeta potential, which became less negative (~−18 mV), when the extract was loaded. The mean diameter of empty glycerosomes and glyhyalurosomes were smaller (~130 nm) and the zeta potential was less negative (~−16 mV) than that of liposomes and hyalurosomes due to the presence of glycerol. The last has a different dielectric constant respect to that of water and can cause a reorganization of both bilayer and vesicle surface. The extract loading led a decrease of the hyalurosome mean diameter in comparison to that of the empty ones (~145 nm, *p* < 0.05 versus the size of corresponding empty vesicles), as hyaluronate may favour a better dispersion of the different components of the extract in the hydrophilic medium of vesicles and cause a different packing of the phospholipid. Indeed, vesicles were smaller and their zeta potential was slightly less negative. The mean diameter of glyhyalurosomes was not affected by the loading of the extract (~135 nm, *p* > 0.05 versus the size of corresponding empty) and the zeta potential was slightly less negative.

*Z. officinalis* loaded vesicles were slightly polydispersed, confirming the cryo-TEM observation. Zeta potential was affected in different ways: the charge of liposomes and hyalurosomes were less negative after the incorporation of *Z. officinalis* extract, changing from ~−67 and ~−55 mV to ~−15 and ~−23 mV, respectively. On the other side, surface charge of glycerosomes and glyhyalurosomes, was more negative, changing from ~−16 mV to ~−26 mV. The entrapment efficiency of liposomes was the lowest (~59%) and significantly improved for the other vesicles without significant differences among these samples (~75%, *p* > 0.05 among the values of glycerosomes, hyalurosomes and glyhyalurosomes).

**Figure 3:**
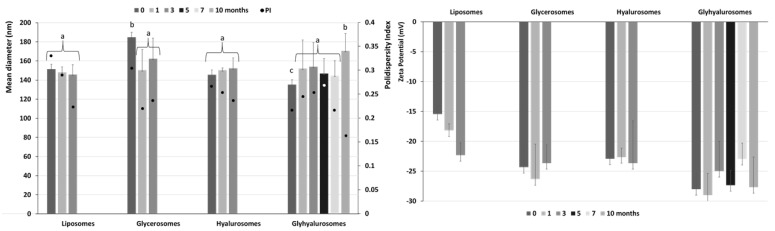
Mean diameter (bars), polydispersity index (dots) and zeta potential, of the extract loaded vesicles stored at 4 °C for 10 months. Data represent the means ± SD of at least six replicates. Each symbol (a, b, c) indicates the same value of mean diameter.

The long-term stability of the formulations was evaluated by storing the samples for a period of 10 months at 4 °C and measuring their physicochemical characteristics (Figure 3). After 3 months the vesicle formulations kept their characteristics as their main parameters tested were almost unchanged. Glycerosomes underwent a significant decreased in size and polidispersity index in comparison to the initial time (*p* < 0.05). Only glyhyalurosomes preserved the physicochemical characteristics for all the storage period, maintaining their physico-chemical characteristics up to 10 months (~170 nm, polydispersity index ~0.16, *p* < 0.05). On the contrary, the mean diameter and zeta potential of liposomes, glycerosomes and hyalurosomes became undeterminable after 3 months. This could indicate that the combination of PEG, glycerol and sodium hyaluronate promoted the formation of a stable vesicle dispersions, where *Z. officinalis* extract is effectively incorporated (Figure 3).

### 3.2. Determination of Size Distribution of Sprayed Droplets

The feasibility of our formulations as nasal spray was evaluated by the analysis of the droplet size distribution by laser diffraction technology (Table 2). The behaviour of vesicle dispersions was very similar: at a distance of 4 cm, the average droplet size of liposomes and hyalurosomes was ~58 µm for D50 and ~121 and ~146 µm for D90 respectively, and that of glycerosomes and glyhyalurosomes was ~78 or ~70 µm for D50 and ~177 or ~160 µm for D90 respectively. At 7 cm, the droplet size decreased slightly, liposomes, hyalurosomes and glyhyalurosomes were ~60 µm for D50 and ~109 µm for D90, glycerosomes was higher, ~68 µm for D50 and ~140 µm for D90. The obtained values of the tested dispersions were comprised between 30–70 µm for D50, and <200 µm for D90, confirming their suitability for nasal local delivery in accordance with the FDA recommendations [38]. Their efficacy was also suggested by the almost complete absence of droplet sizes <10 µm.

### 3.3. Measurements of Spray Plume Morphology and Angle

#### Instant Views

**Figure 4:**
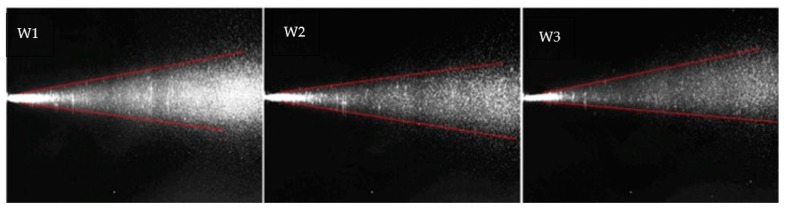
Representative images of instant visualization of the subsequent three water sprays.

The spatial distribution of the sprays was determined from instantaneous images. To assess the possible variability introduced by pump and nozzle manufacture, the behaviour of distilled water sprayed different times by the same device was evaluated as well, and used as reference (Figure 4). The sprays of water generated full narrow cones of drops (plume) remarkably similar in dimension and geometry and only break up length was larger for sprays W2 and W3.

Observation confirmed that the three sprays are nearly identical, and the only visually appreciable difference was that the cone angle of spray W1 was slightly wider in comparison with those for sprays W2 and W3. So, appreciable differences were due to the atomizer pump can be neglected and all the obtained results for the different formulations are reliable.

**Figure 5:**
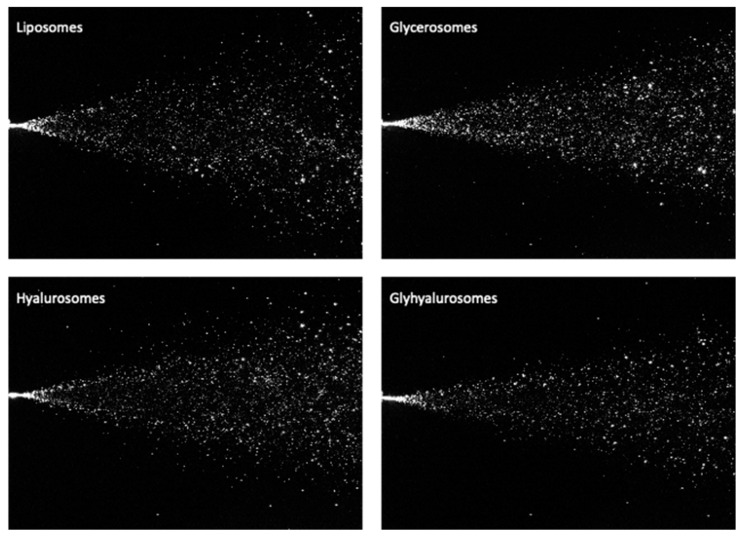
Representative images of instant visualization of sprays generated by liposomes, glycerosomes, hyalurosomes and glyhyalurosomes.

The atomization cone of vesicle dispersions was larger than that of water and the drops were homogenously distributed on the generated cone sprays (Figure 5).

In particular liposomes and hyalurosomes generated a more dispersed nebulization, were droplets spread around the generated cone, which was wider and less dense in drops. The plume of glycerosomes and glyhyalurosomes was more compact and homogeneous, with a narrower plume angle respect to the other formulations. The spray angle was measured to better evaluate the differences among the formulations (Table 3). The angle of the cone generated by glycerosomes and glyhyalurosomes was around 22° while that of liposomes and hyalurosomes was around 30°, indicating the contribution of glycerol on the formation of homogeneous and narrow cone probably due to the increased viscosity of the dispersion. The small angle can facilitate the deposition of drops in the anterior part of the nasal cavity [42,43].

### 3.4. Average Velocity Module

The velocity modulus of sprayed formulations was calculated averaging 100 instantaneous measurements. The resulting images of velocity modulus provide information of the structure of the semi-hollow cone spray.

**Figure 6:**
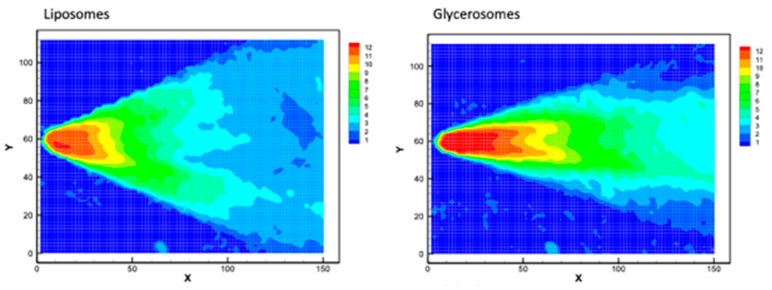

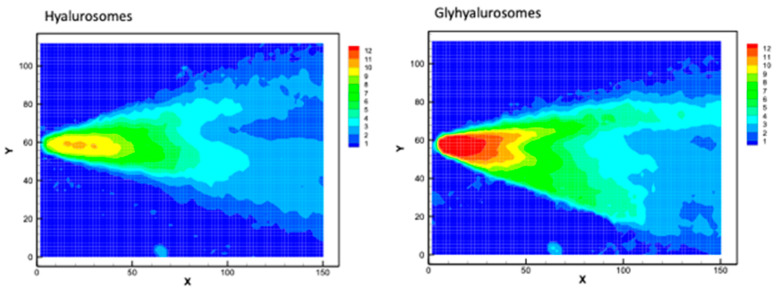
Images of modulus of average velocity of sprayed liposomes, glycerosomes, hyalurosomes and glyhyalurosomes.

Figure 6 shows the mean velocity of the droplets along the midplane of the spray. The spray speed of drops of hyalurosomes was the lowest (~10.1 m/s) while that of liposomes was higher (~12.2 m/s) followed by that of glycerosomes and glyhyalurosomes (~13.15 m/s). All the atomizers generate hollow-cone sprays, but the spray angle was diminished when glycerol was added, most likely due to an increase of the liquid viscosity, as already pointed out. In addition, the area of large absolute velocity (in red in the figures) becomes thinner and larger for the formulations with glycerol (see also Table 3). These data suggest that the increase of the viscosity worsen the performance of the atomization device, which is a usual behaviour of almost all atomizer families. However, the maximum velocity level (Table 3) is similar for all formulations except when the device atomized just hyalurosomes. A possible explanation to this exception could be a slightly defective atomizer device. Although the present atomizers can atomize reasonably well the liquids, such viscous formulations are not easy to be atomized and they require devices without any fault, even the slightest.

### 3.5. Biocompatibility of Vesicles and Protective Effect against Oxidative Stress Damage

**Figure 7:**
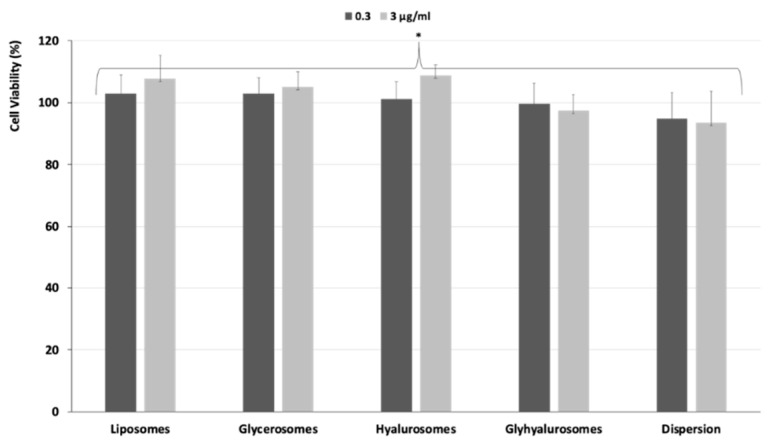
Viability of keratinocytes treated with *Z. officinalis* extract in dispersion or loaded in liposomes, glycerosomes, hyalurosomes and glyhyalurosomes and properly diluted to reach two different concentrations (0.3, 3 µg/mL of extract). Data represent the means ± standard deviation of at least three experimental determinations. The symbol (*) indicates the same value (*p* > 0.05).

The biocompatibility of *Z. officinalis* loaded vesicles was evaluated using human epithelial cells. The cells were incubated with the formulations for 48 h and their viability was measured (Figure 7). The dispersion of the *Z. officinalis* extract in water was used as reference. The cell viability after incubation with dispersion and vesicle formulations was >90% irrespective of the used concentration (0.3, 3 µg/mL) (*p* > 0.05 among all the values).

**Figure 8:**
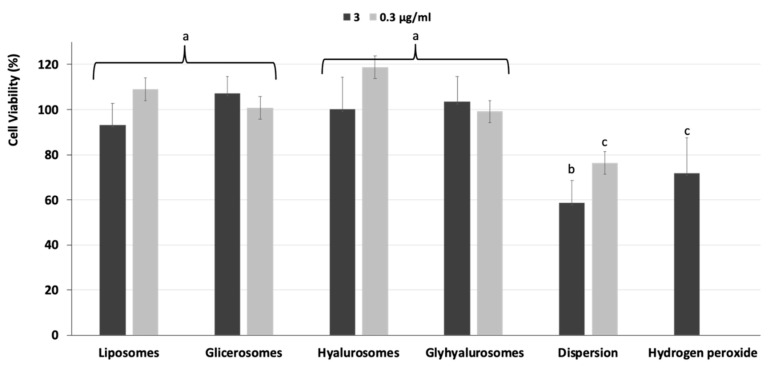
Viability of epithelial cells stressed with hydrogen peroxide and treated with ginger extract in dispersion or loaded in liposomes, glycerosomes, hyalurosomes and glyhyalurosomes. Data are reported as mean values ± standard deviation of cell viability expressed as the percentage of the negative control (100% viability). Each symbol (a, b, c) indicate the same value.

The ability of *Z. officinalis* extract loaded vesicles to scavenge the peroxide radicals generated by hydrogen peroxide, thus protecting the cells from damage and death, was also evaluated (Figure 8). The stressing with hydrogen peroxide decreased the cell viability up to 70%. The treatment with extract in dispersion using the higher concentration of extract (3 µg/mL) was not effective and cell viability further decreased (<60%, *p* < 0.05 versus the viability of cells stressed with hydrogen peroxide). On the contrary, the treatment with extract loaded in vesicles provided an effective protection from oxidative damages avoiding or reducing the cell death. Indeed, the viability of cells treated with liposomes, glycerosomes, hyalurosomes and glyhyalurosomes was ≥100% irrespective to the used dilution. Only cells treated with liposomes loading 3 µg/mL showed a slightly lower viability (~90%). These data suggest an effective ability of vesicles to improve the efficacy of the extract and to counteract the damages induced in cells by oxidative stress.

## 4. Discussion

In the present study, aiming at manufacturing green and natural nanoformulations suitable for the treatment of rhinitis and rhinosinusitis, the *Z. officinalis* extract was loaded in hyalurosomes modified with glycerol [44,45]. Each component was selected based on its beneficial properties on nasal cavity. Indeed, phospholipids, aggregated as lamellar vesicles, strength the barrier function of nasal mucosa, which play an important role in the patho-mechanism of the allergic rhinitis [13]. In a previous study, 60 patients with chronic rhinosinusitis were treated with liposomal nasal spray and 30 patients with steroid-based therapy. The treatment with liposomal nasal spray resulted in a similar reduction of symptoms and a significant improvement of the quality of life, confirming their suitability as valuable alternative intended for nasal administration [14]. Hyaluronan exerts hydrating and lubricating activities, plays a role in controlling inflammatory airway processes and mucociliary clearance, and it is also involved in tissue healing and remodeling [46]. Indeed, its nasal solution was able to significantly reduce symptoms of chronic rhinosinusitis in human patients [44]. In pharmaceutical applications, glycerol has an hydrating and protective effect on skin and *mucosae*, promotes the wound-healing processes and has an antibacterial effect [45,46,47,48,49,50]. These properties are essential in topical formulations, such as dermal or nasal preparations, to enhance their therapeutic effect. Moreover, glycerol is water-miscible and stable in aqueous phase, thanks to the formation of intramolecular hydrogen bonds [51,52]. Its hygroscopic characteristics, given by three hydrophilic hydroxyl groups, improves its smoothness and humectant capacity [45]. Finally, the extract of *Z. officinalis* has been used as a plant-derived therapeutic date back to more than 2000 years ago. *Z. officinalis* based remedy preparations can be read in Greek, Roman, Arabic and Buddhist medical literature [53,54]. Its anti-inflammatory action was already known to this populations, especially to counteract viral infections and colds [11]. Nowadays many authors are confirming the anti-inflammatory, antioxidant, anti-bacterial and analgesic efficacy of *Z. officials* and it is still widely used as medical plant in therapeutic preparations [55,56]. Recent research studies confirmed its anti-inflammatory and antioxidant effects especially due to several bioactive components such as gingerol (5%), shogaol, paradol, isogingerol, isoshogaol, gingeridone, quercetin and catechin [57,58,59,60]. The combinations of these components is expected to counteract the mucosal irritation and dryness typically present in rhinitis and rhinosinusitis [61]. In addition, the traditional therapy based on plant-derived bioactives has been improved by its combination with advanced pharmaceutical nanotechnology by means of a totally green and environmentally friendly approach [62]. Thus, only natural occurring components were used: soy phosphatidylcholine, *Z. officinalis* extract, hyaluronan, glycerol and water. Moreover, vesicles were prepared by direct sonication, which is an easy and low dissipative method, which can be scaled up at industrial level [63]. The resulting vesicles were sized around 150 nm and had an oligolamellar and multicompartment structure, which as reported in a previous study, improved the payload delivery inside the biological membrane [64]. Glyhyalurosomes were the only stable formulation as their physicochemical characteristics remained unchanged during the 10 months of storage, while the other ones aggregated and fused already after 3 months.

To quantify the deposition of the generated droplets in the target regions represents a critical step especially for the development of nasal products, as in that case it mainly depends of size and velocity of droplets and plume angle along with nasal anatomy and inter-subject variability related to age, gender, and ethnicity among human individuals [65,66]. The size distribution of sprayed droplets, generated from the different formulations, was between ~60 and ~180 µm, suggesting their feasibility and ability to reach the anterior nasal cavity after spray, in accordance with the FDA guidelines [67], and exerting a local effect on the nasal mucosae. All the vesicle dispersions, especially glycerosomes and glyhyalurosomes were atomized in fine droplets homogenously distributed in a full cone plume, with an angle ranging from 20 to 31°. The angle of plume generated by glycerosomes and glyhyalurosomes was narrower than that of liposomes and hyalurosomes, probably due to the presence of glycerol, which led to the formation of a more viscous system. A narrow plume with an angle < 30° is not the most effective for the deposition of the formulation in the anterior part of the nose and can favour the deposition in the turbinate region as a function of the droplet size and velocity [65,68,69,70]. Indeed, droplets with high-speed are deposited in the anterior part of the nose [70]. Thus, despite the small plume angle, the size and velocity of droplets generated by glycerosomes and glyhyalurosomes can ensure a good deposition in the anterior part of the nose because of their high kinetic energy that may result in a considerable inertial deposition on the nasal surfaces close to the spray inlet [41]. Deposition efficiencies of approximately 90% could be achieved modifying the degrees, such as using 30° administration angles. After the drop deposition in the nasal mucosa, *Z. officinalis* extract loaded hyalurosomes and glyhyalurosomes can effectively exert its effects strengthening the barrier function and hydrating, lubricating and moisturizing the mucosa. This effect is very important because nasal mucus is the first line defence barrier against a variety of inhaled pathogens. With around 12,000 L of air inhaled daily, the airways may enter in contact with 25 million particles per hour that need to be filtered and/or transported away [1,2,3]. The barrier protection exerted by vesicles has been further strengthened by the antioxidant protection of the extract. Indeed, the loading of the extract into the vesicles maximize its efficacy, as vesicles were capable of counteracting the damages induced by oxidative stressed with hydrogen peroxide, avoiding the death of cells and maintaining their viability ~100% even after injury. Thus, glycerosomes and hyalurosomes are the most suitable formulations for nasal delivery mainly because of compactness of the generated cone (cone angle between ~20 and 24°), droplets size (comprised between ~70 and 180 µm), and a high-speed (~13 m/s). Between these, glyhyalurosomes, which further contain hyaluronic acid, seem to be the most promising formulation for the treatment of local diseases such as nasal allergic conditions and nasal congestions thanks to their high stability during the whole storage period and to their capability to protect the nasal cavity.

## 5. Conclusions

The use of *Z. officinalis* extract passes through millennia and nowadays its efficacy continues to be validated by modern scientific research. The incorporation of *Z. officinalis* extract in hyalurosomes enriched with glycerol, allows to obtain a formulation based on natural components, prepared by green method, stable on storage, effective as antioxidant, which can be sprayed in the anterior part of the nasal cavity. The obtained glyhyalurosomes, being the most promising formulation should be further evaluated for the manufacture of natural and green nasal spray for the prevention and treatment of rhinitis and rhinosinusitis.

## Figures and Tables

**Table 1:** Mean diameter (MD), polydispersity index (PI), zeta potential (ZP), and entrapment efficiency (EE) of the empty and extract loaded vesicles. Data represent the means ± SD of at least six replicates. Each symbol (a, b, c, d, e, f) indicates the same value of mean diameter (*p* > 0.05).

	MD (nm ± SD)	PI	ZP (mV ± SD)	EE%
**Empty liposomes**	^c^ 92 ± 2	0.28	−67 ± 4	
**Empty glycerosomes**	^a^ 128 ± 13	0.39	−15 ± 4	
**Empty hyalurosomes**	^d^ 175 ± 25	0.35	−55 ± 7	
**Empty glyhyalurosomes**	^a^ 132 ± 10	0.39	−18 ± 1	
**Ginger liposomes**	^a^ 151 ± 1	0.33	−15 ± 1	^b^ 59 ± 2
**Ginger glycerosomes**	^d^ 185 ± 8	0.30	−24 ± 3	^e^ 88 ± 4
**Ginger hyalurosomes**	^f^ 145 ± 1	0.26	−23 ± 1	^e^ 67 ± 13
**Ginger glyhyalurosomes**	^a^ 135 ± 9	0.21	−28 ± 2	^e^ 72 ± 10

**Table 2:** Droplet size analysis of *Z. officinalis* vesicles sprayed from a distance of 4 and 7 cm from the laser beam. Standard deviation (±) is expressed as the mean of 3 measurements. Each symbol (a, b, c, d, e, f) indicates the same value.

		4 cm				7 cm		
	D10 (µm)	D50 (µm)	D90 (µm)	SPAN (µm)	D10 (µm)	D50 (µm)	D90 (µm)	SPAN (µm)
**Liposomes**	26 ± 1	^a^ 57 ± 4	^c^ 121 ± 14	1 ± 0.1	32 ± 2	^a^ 58 ± 2	^c^ 109 ± 3	1 ± 0.05
**Glycerosomes**	30 ± 0.7	^b^ 78 ± 2	^d^ 177 ± 3	2 ± 0.00	35 ± 1	^f^ 68 ± 2	^e^ 141 ± 3	1 ± 0.1
**Hyalurosomes**	24 ± 1	^a^ 59 ± 4	^e^ 146 ± 16	2 ± 0.1	36 ± 2	^a^ 61 ± 3	^c^ 109 ± 3	1 ± 0.1
**Glyhyalurosomes**	27 ± 0.3	^f^ 70 ± 4	^e^ 160 ± 11	2 ± 0.04	36 ± 1	^a^ 60 ± 1	^c^ 103 ± 9	1 ± 0.1

**Table 3:** Average maximum velocities and spray angle generated by liposomes, glycerosomes, hyalurosomes and glyhyalurosomes.

	Liposomes	Glycerosomes	Hyalurosomes	Glyhyalurosomes
**Max velocity (m/s)**	12.2	13.2	10.1	13.1
**Spray angle (°)**	31.1	20.0	27.7	24.4

## Data Availability

Data is contained within the article.
